# Lymph Node Biopsy Specimens and Diagnosis of Cat-scratch Disease

**DOI:** 10.3201/eid1209.060122

**Published:** 2006-09

**Authors:** Jean-Marc Rolain, Hubert Lepidi, Michel Zanaret, Jean-Michel Triglia, Gérard Michel, Pascal-Alexandre Thomas, Michèle Texereau, Andreas Stein, Anette Romaru, François Eb, Didier Raoult

**Affiliations:** * Université de la Méditerranée, Marseille, France;; † Fédération Oto-Rhingo-Laryngologie, Marseille, France;; ‡ Hôpital Timone Enfant, Marseille, France;; § Hôpital d'Enfants de la Timone, Marseille, France;; ¶ Hôpital Sainte-Marguerite, Marseille, France;; # Fédération de Médecine, Niort, France;; ** Hôpital de la Conception, Marseille, France;; †† Laboratoire de Biologie, Niort, France;; ‡‡ Centre Hospitalier Universitaire, Amiens, France

**Keywords:** Bartonella, lymphadenopathy, cat-scratch disease, 16S rRNA, mycobacteria, research

## Abstract

We report microbiologic analysis of 786 lymph node biopsy specimens from patients with suspected cat-scratch disease (CSD). The specimens were examined by standard, cell culture, and molecular methods. Infectious agents were found in samples from 391 (49.7%) of 786 patients. The most commonly identified infectious agent was *Bartonella henselae* (245 patients, 31.2%), the agent of CSD. Mycobacteriosis was diagnosed in 54 patients (6.9%) by culture and retrospectively confirmed by using a specific real-time PCR assay. Neoplasm was diagnosed in 181 specimens suitable for histologic analysis (26.0%) from 47 patients. Moreover, 13 patients with confirmed *Bartonella* infections had concurrent mycobacteriosis (10 cases) or neoplasm (3 cases). A diagnosis of CSD does not eliminate a diagnosis of mycobacteriosis or neoplasm. Histologic analysis of lymph node biopsy specimens should be routinely performed because some patients might have a concurrent malignant disease or mycobacteriosis.

## Abstract 2

Histologic analysis of lymph node biopsy specimens may verify diagnosis of this disease.

Lymph node enlargement is a common medical problem. Infections caused by bacterial, viral, and protozoal agents are the most typical cause of localized lymphadenopathy, but malignancies or lymphoproliferative diseases are also often found (*1*). Physicians must differentiate malignant lymphadenopathies or infectious diseases that require special care from benign reactive lymphadenopathy or self-limiting adenitis.

In a large number of patients, the causes of lymphadenopathy remain undiagnosed. Causes of lymphadenopathy other than neoplasm that require urgent medical attention include tuberculosis and HIV infection. During the past 15 years, *Bartonella henselae*, the causative agent of cat-scratch disease (CSD), has been reported as a common cause of localized lymphadenopathy (*1**–**3*). Diagnostic techniques for *Bartonella*-related infections include culture of the pathogen (*4**,**5*), detection of organisms in lymph nodes by immunofluorescence (*6*), molecular techniques including PCR amplification of *Bartonella* spp. genes (*7**,**8*), and serologic analysis (*9**,**10*). *B*. *henselae* is not commonly isolated from CSD patients (*4**,**11*), and PCR-based detection of various target genes of *Bartonella* species in tissue specimens has become the most widely accepted way of diagnosing CSD (*7**,**8*).

Serologic analysis is a minimally invasive diagnostic technique that has been extensively evaluated for the diagnosis of CSD (*9**,**10**,**12*). The sensitivity of serologic tests varies from 1 laboratory to another, ranging from nearly 100% to <30% (*9*). Specificity may also vary, and a specificity >95% may be achieved by using commercial tests with immunoglobulin G cutoff titers >128 (*10*).

As a national reference center for rickettsioses and bartonelloses, we routinely receive lymph node biopsy specimens from patients with suspected CSD. In this study, we analyzed a large collection of lymph node biopsy samples obtained from January 2001 through August 2005 using microbial cultures (blood agar culture and cell culture) and 16S rDNA- and *Bartonella*-specific PCR. Our objective was to define the frequency of *B*. *henselae* and other bacterial infections in patients with suspected CSD in France.

## Methods

### Patients

We studied lymph node biopsy specimens from patients with suspected CSD that were collected from January 2001 through August 2005. Tissues specimens sent to our reference center were obtained from both hospitalized patients and outpatients throughout France. We receive either the entire lymph node or a fragment of it; the specimens were sent either frozen or in transport media. This factor is crucial because most of the specimens received were not in suitable condition for histologic analysis. A definitive diagnosis of CSD was defined as a biopsy sample that was positive by PCR for 2 different target genes of *Bartonella* spp (*6*). If a specimen had been previously analyzed and *B*. *henselae* was reported (*7*), the specimen was excluded from the present study.

### Detection of *Bartonella* DNA in Tissue Specimens

Total genomic DNA was extracted from samples with a QIAamp tissue kit (Qiagen, Hilden, Germany) as previously described (*7*). Samples were handled under sterile conditions to avoid cross-contamination. Genomic DNA was stored at 4°C until used as template in PCR assays. The primers used for *B*. *henselae* amplification and sequencing (internal transcribed spacer [ITS] region and *pap31* gene) have been previously evaluated (*6**,**7*). Up to 10 samples were tested, along with negative controls (DNA from noninfected lymph nodes and sterile water) and a positive control (DNA from *B*. *elizabethae* for the ITS region, GenBank accession no. L35103, and DNA from *B*. *henselae* Houston-I for the *pap31* gene, GenBank accession no. AF001274).

### Detection of Bacteria in Tissue Specimens

Nucleic acids were extracted with a QIAamp tissue kit (Qiagen) and PCR performed with universal 16S rDNA primers fD1 and rp2 (Eurogentec, Seraing, Belgium) (*13*) and Taq DNA polymerase (GIBCO-BRL Life Technologies, Gaithersburg, MD, USA). Amplification and sequencing of products were conducted as previously described (*14*). Up to 10 samples were tested, along with negative controls (noninfected lymph node and sterile water) and positive controls (*B*. *henselae* Houston-I and *Staphylococcus aureus* (ATCC 29213). The 16S rDNA sequences obtained were compared with all bacterial 16S rRNA sequences available in the GenBank database by using the Blastn version 2.2.2 program (National Center for Biotechnology Information, Bethesda, MD, USA). The efficiency of DNA extraction and presence of inhibitors in samples that were negative by PCR were tested by using primers that targeted a fragment of the human β-globin gene as previously described (*15*).

### Detection of *B. henselae* in Lymph Nodes

We confirmed *B*. *henselae* in lymph nodes of patients with CSD by using a specific monoclonal antibody for *B*. *henselae* as previously described (*6*). The slides were air-dried and fixed with methanol for 10 minutes at room temperature before testing with an immunofluorescence assay (*6*). The sensitivity and specificity of this assay and antibody were previously reported to be 79.6% and 92.5%, respectively (*6*).

### Culture Methods

Lymph node biopsy specimens were placed on blood agar plates, incubated at 37°C in an atmosphere of 5% CO_2_, and examined weekly for growth during a 2-month period. This process resulted in isolation of either *Bartonella* or mycobacteria (*16*). Specimens were also placed on human embryonic lung cells in shell vials and incubated at 37°C in an atmosphere of 5% CO_2_ as previously described (*4**,**17*). From January 2002 to August 2005, specimens were also incubated onto horse blood agar supplemented with hemin (100 mg/L). This procedure has been reported to improve the isolation rate of *B*. *henselae* and can also support growth of rapidly growing mycobacteria (*11**,**16*). Specimens were also cultured under anaerobic conditions. *Bartonella* isolates were identified by PCR and sequencing as described above; other bacterial isolates were identified by using standard bacteriologic methods. Samples from which mycobacteria were isolated were reanalyzed retrospectively by real-time PCR with modified primers and probes targeting the ITS region as previously described (*18*).

### Histologic Analysis

Samples that had not been frozen (181 specimens) were fixed in formalin and processed for histologic analysis. Stains used included Gram, hematoxylin and eosin, periodic acid–Schiff, Ziehl-Neelsen, and Warthin-Starry.

### Statistical Analysis

Two groups of patients were defined for demographic data comparisons: CSD patients (detection of *Bartonella* DNA) and non-CSD patients (no detection of *Bartonella* DNA). For data comparison, the Student *t* test or χ^2^ test was performed by using EpiInfo version 6.0 software (Centers for Disease Control and Prevention, Atlanta, GA, USA).

## Results

### Diagnoses in Patients with Lymphadenopathy

We tested 786 lymph node biopsy specimens from patients with suspected CSD. Only 181 specimens were suitable for histologic analysis. Neoplasm was diagnosed by histologic analysis in 47 (26.0%) of 181 patients (6 with skin carcinomas, 1 with acute leukemia, 24 with lymphomas, 12 with Hodgkin disease, and 4 with Kaposi sarcoma). Bacteria were cultured from 143 specimens (18.2%), and mycobacteria were the most frequently recovered organisms (54 [6.9%] of 786) on blood agar or by shell vial culture (Table 1). The 54 nodes that contained mycobacteria were retrospectively confirmed by using real-time PCR targeting the ITS region. Other common bacteria recovered either by culture or PCR were staphylococci (26 cases) and *Propionibacterium acnes* (15 cases). *B*. *henselae* was cultured and successfully passaged from 1 lymph node, and *B*. *quintana* was cultured and amplified from 1 lymph node. Fastidious bacteria were cultured from lymph nodes by the shell vial cell culture: 2 isolates of *Coxiella burnetii* and 1 isolate of *Francisella tularensis*, which has been previously reported (*19*) (Table 1). Anaerobic bacteria cultured from lymph nodes included *Fusobacterium* spp. (4 specimens), *Prevotella* sp. (1 specimen), and *Clostridium perfringens* (1 specimen).

**Table 1:** Results of culture and PCR assays of 786 biopsy lymph node specimens*

Diagnosis or infection	Positive culture	*Bartonella*- positive PCR	16S rDNA–positive PCR	Total
CSD	1	244	122	244
*Bartonella quintana*	1	1	1	1
Q fever	2	0	3	3
Tularemia	1	0	1	1
*Abiotrophia adjacens*	2	0	2	2
*Actinomyces*	1	0	1	1
*Pasteurella multocida*	2	0	2	2
Mycobacterial infection	54	0	32	54
*Staphylococcus aureus*	16	0	16	16
Coagulase-negative *Staphylococcus*	15	0	10	23
*Streptococcus pyogenes*	10	0	10	10
*Fusobacterium* spp.	4	0	4	4
*Nocardia asteroïdes*	1	0	1	1
*Propionibacterium acnes*	15	0	7	16
*Prevotella* sp.	1	0	1	1
*Clostridium perfringens*	1	0	1	1
*Tropheryma whipplei*	0	0	1	1
Miscellaneous	21	0	21	21
Neoplasm	0	0	0	47
Unknown	0	0	0	350
Total	148	245	236	449

*CSD, cat-scratch disease. Among 244 specimens PCR-positive for *B. henselae*, 10 showed a concurrent mycobacterial infection and 3 showed a neoplasm.

Amplification of the 16S rDNA gene for common bacteria was performed on all specimens. Positive results were obtained for 236 patients (30.0%), and *B*. *henselae* was the most frequently amplified bacterium (122 cases, 51.7%). Other bacteria commonly detected included mycobacteria, staphylococci, streptococci, and *P*. *acnes* (Table 1). Fastidious bacteria were isolated from 5 lymph nodes: *C*. *burnetii* (3 cases), *F*. *tularensis* (1 case), and *Tropheryma whipplei* (1 case). These 5 diagnoses were confirmed by a second specific PCR with primers and probes routinely used in our laboratory. Using specific primers for the ITS region and *pap31* gene of *Bartonella* spp., we identified *Bartonella* spp. in 245 patients (31.2%), including 122 patients identified by PCR with primers for the 16S rDNA gene. No discordance was observed between the ITS region and the *pap31* gene.

When compared with specific detection of *Bartonella* DNA, specificity of the 16S rDNA PCR was 100% but sensitivity was low (49.8%, 122 of 245 lymph nodes were positive). Positive and negative controls showed expected results in all tests. All but 1 of the sequences of the ITS region and *pap31* genes we obtained were identical to those of *B*. *henselae* reported in GenBank. In 1 patient, the sequences obtained were identical to those of *B*. *quintana*. Among these 245 samples positive for *Bartonella*, 216 were also tested by direct immunofluorescence assay with monoclonal antibodies to *B*. *henselae*, of which 166 (76.9%) were positive.

A total of 391 (49.7%) of 786 patients had an infectious disease (including the 10 patients whose specimens were *B*. *henselae*–positive by PCR and showed mycobacterial infection), 47 had neoplasm (including 3 specimens with *B*. *henselae*–positive PCR result), and 351 (44.6%) had no identified cause for their lymphadenopathy (Table 1). On the basis of these results, we divided the patients into 2 groups: patients with a positive PCR result for *Bartonella* (n = 245) (CSD group) and the remaining patients (n = 541) (non-CSD group).

### Comparison of Demographic Data between Patient Groups

**Figure 1:**
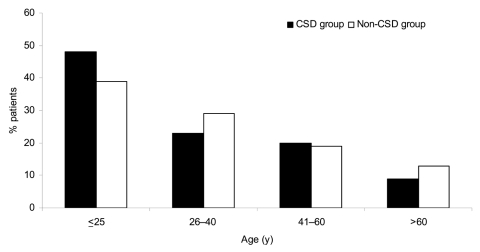
Distribution of patients by age and group. Cat-scratch disease (CSD) group, patients with Bartonella-positive PCR results in lymph node samples; Non-CSD group, patients with Bartonella-negative PCR results. For patients <25 years of age, p = 0.032 for CSD group versus non-CSD group.

The mean ± standard deviation (SD) age was 30.2 ± 20.4 years (range 1–94 years) in 245 patients with proven *B*. *henselae* or *B*. *quintana* lymphadenopathy (CSD group) versus 31.6 ± 20.7 years (range 4 months to 86 years) in the non-CSD group. Most patients with *B*. *henselae* CSD were <25 years of age (p = 0.032) (Figure 1). The mean ± SD ages of patients with neoplasm (46.2 ± 22.6 years, range 7–86 years) and mycobacterioses (39.5 ± 22.2 years, range 1–84 years) were greater than the mean ± SD age of patients with CSD (p<0.05 by Student *t* test) (Table 2). The sex ratio (male:female) was 1.28 in the CSD group and 1.50 in the non-CSD group, but this difference was not significant (p>0.05) (Table 2). In the CSD group, 89 of the lymph node biopsy specimens were from axillary nodes (36.3%), 75 were from inguinal nodes (30.6%), and 81 were from cervical nodes (33.1%).

**Table 2:** Comparison of demographic data between CSD patients and non-CSD patients*

Factor	No. patients	Age, y (mean ± SD)	Sex ratio (M/F)	p value†
CSD group (total)	245	30.2 ± 20.4	1.28	
CSD alone	231	29.4 ± 19.6	1.26	
CSD plus mycobacteria	10	43.3 ± 8.2	1.0	
CSD plus neoplasm	3	57.3 ± 6.0	3.0	
*Bartonella Quintana* alone	1	31.6 ± 20.7		
Non-CSD group	541	39.5 ± 22.2	1.50	>0.05
Mycobacteria	44	46.2 ± 22.6	1.72	<0.05
Neoplasm	44	30.2 ± 20.4	1.30	<0.05

*CSD, cat-scratch disease; SD, standard deviation. †Comparison of mean age of CSD group and corresponding non-CSD group.

**Figure 2:**
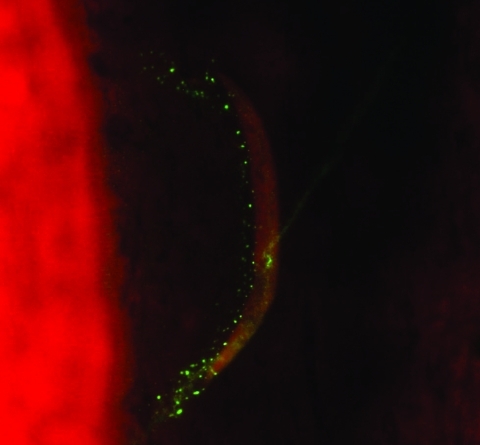
Detection of Bartonella henselae in the lymph node of a patient with cat-scratch disease and tuberculosis by direct immunofluorescent assay with a monoclonal antibody (magnification ×400).

We found that 13 of 245 patients with CSD had concurrent lymph node disease (Table 2). Ten had mycobacteriosis proven by culture (5 with *M*. *tuberculosis*, 3 with *M*. *avium*, 1 with *M*. *fuerthenensis*, and 1 with *M*. *gordonae*), and 3 had neoplasm (2 with lymphoma and 1 with Hodgkin disease). The mean ± SD age of these 13 patients (49.7 ± 16.0 years, range 27–72 years) was higher than the mean ± SD age of the remaining 232 patients with only CSD (p<0.05 by Student *t* test). Only 4 lymph node biopsy specimens from the 10 patients with concurrent mycobacteriosis were positive by Ziehl-Neelsen staining. Six of 10 lymph node biopsy specimens were positive in a direct immunofluorescence assay with monoclonal antibodies for *B*. *henselae* (Figure 2) as previously described (*6*).

Of the 3 patients with CSD and concurrent neoplasm, a positive PCR result for the 16S rDNA gene was obtained with DNA from 1 lymph node (*B*. *henselae*). Two of 3 lymph nodes were positive in a direct immunofluorescence assay with monoclonal antibodies to *B*. *henselae* as described previously (*6*). As expected, the number of patients with either mycobacteriosis or neoplasm in the non-CSD group was higher than in the CSD group (p = 0.014; n = 181 patients).

## Discussion

Culture and PCR were used to examine lymph node biopsy specimens from patients with suspected CSD. These methods, i.e., blood agar and cell culture (*20*), molecular biology with PCR for the 16S rDNA gene (*14*), PCR with 2 specific genes from *Bartonella* (*6**,**7*), and histologic analysis (*20*), have been previously validated and are routinely used for examination of lymph node samples. Our report describes an extensive study on lymph nodes using culture, 16S rDNA PCR amplification, and amplification of target genes of *Bartonella* spp.

Our objective was to define all bacterial causes of lymphadenopathies for samples initially sent to our center for detection of CSD. In the patients we studied, 50% had infectious diseases, and the most common causative agent was *B*. *henselae*; ≈30% of suspected patients were PCR positive (CSD group). Sensitivity of PCR with the 16S RNA gene to diagnose CSD was lower than was *Bartonella*-specific PCRs. The sensitivity of PCR assays with the 16S rRNA gene for the diagnosis of CSD has been reported to vary from 43% to 100%, depending on the primers used and the definition of a positive case (*21**,**22*). In our laboratory, PCR with specific primers against *Bartonella* genes is more sensitive and specific in the diagnosis of CSD.

In a recent study in Germany, *B*. *henselae* was the causative agent of head and neck lymphadenopathy in 61 (13.4%) of 454 patients (*1*). As in our study, *B*. *henselae* was the most common organism responsible for lymphadenopathy in adults and children (*1*). However, our higher percentage of positive PCR results was because specimens sent to our reference laboratory were from patients with suspected CSD. Many cases of CSD remain unrecognized because serologic or molecular analyses are not routinely used. We observed a low isolation rate for *B*. *henselae* on axenic media or in cell culture, only 1 successfully passaged isolate among the 245 PCR-positive samples, which is consistent with previous findings (*4**,**8*). This rate did not improve when we used an enriched medium designed to improve isolation of *B*. *henselae* (*11*). A recently developed enriched liquid medium for growth of *Bartonella* strains (*23*) may be useful in obtaining more isolates of *B*. *henselae* from patients with CSD. However, in many lymph nodes negative by culture, we observed bacteria by direct immunofluorescence, which suggests that bacteria in lymph nodes are not viable (*6*). Consistent with this finding was that most nodes were necrotic at histopathologic examination (data not shown). One lymph node was positive for *B*. *quintana* by culture and PCR as previously reported (*24*).

The long incubation time needed for isolation of *Bartonella* allows us to isolate mycobacterial strains by using blood agar culture (*16*). We found mycobacteria incidentally and not because of a specific search. Moreover, even if mycobacteria grew well in blood agar plates (*16*), sensitivity of culture from lymph nodes is not 100%. This fact means that the percentage of mycobacterial infections in our study was probably underestimated because specific PCR for mycobacteria was only performed retrospectively in culture-positive specimens. On the basis of these results, we now routinely perform Ziehl-Neelsen staining and PCR to detect mycobacteria in all specimens.

Before the discovery of *B*. *henselae* and the use of PCR for its diagnosis, mycobacteria were the most frequent infectious agents causing lymphadenopathy (*25*), and staphylococci and group A streptococci were the main causes of acute adenitis. In our study, mycobacteria were the second most common infectious cause of lymph node enlargement; >6.9% of patients were infected. The 16S rRNA PCR in our study had a lower sensitivity than culture in the diagnosis of mycobacterial infection. This finding may have resulted from sample pretreatment to adequately purify DNA (*26*). Freidig et al. found that 24 (5.7%) of 419 lymph nodes were enlarged because of mycobacterial infection (Table 3) (*27*). Similar incidences have been reported by Doberneck (*28*) and Anthony and Knowles (*29*) (Table 3). Higher incidences of mycobacterial infections (27 [16.6%] of 163 lymph node biopsy specimens) were reported by Roberts and Linsey (*25*). In our study, 76% of mycobacterial infections were *M*. *tuberculosis*; 54% were *M. tuberculosis* in the study by Freidig et al. (*27*). This finding is consistent with the fact that the incidence of typical and atypical mycobacterial adenitis is age dependent; typical adenitis is more common in adults, and atypical adenitis is more common in children (*30*).

**Table 3:** Relevant studies of causes of lymphadenopathy, 1983–2004*

Variable	Doberneck (*28*)	Roberts (*25*)	Anthony (*29*)	Freidig (*27*)	Ridder (*1*)	Chau (*36*)	This study
Years	1972–1982	1978–1983	1983	1978–1986	1997–2001	1996–2001	2001–2005
No. patients	169	163	228	419	454	423	786
Mean age, y (range)	34.6 (1–78}	(1–90)	(0–>60)	46.7 (2–89)	34.9 (2–90)	40 (14–90)	32.0 (1–94)
Infectious diseases (%)	8/79 (10.1)	76	11 (4.8)	66 (15.8)	156 (34.4)	75 (17.7)	391 (49.7)
CSD (%)	0	0	3	0	61 (13.4)	3	245 (31.2)
Mycobacteria (%)	5/79 (6.3)	27 (16.6)	6 (2.6)	24 (5.7)	5 (1.1)	12 (2.8)	54 (6.9)
Staphylococci or streptococci (%)	3/79	41	NA	2	13	2	49
Malignant process (%)	119 (70.4)	51 (31.2%)	60 (26.3)	113 (27.0)	52 (11.5)	95 (17.3)	47 (26%)†
Undiagnosed (%)	42 (24.9)	28 (17.2)	68 (29.8)	113 (27.0)	171 (37.7)	168 (39.7)	350 (44.6)

*CSD, cat-scratch disease. †Only 181 samples could be tested by histopathologic analysis.

Other agents found in our study were staphylococci and miscellaneous aerobic and anaerobic bacteria. Isolates of coagulase-negative staphylococci or *P*. *acnes* may be considered contaminants, but the remaining organisms are pathogens and should be considered causative agents of lymph node enlargement (*31*). We found that rare or fastidious organisms may be the cause of infectious adenitis. Such situations have been previously reported, especially infections with *Nocardia* spp (*32*), *C*. *burnetii* (*33*), *F*. *tularensis* (*34*), or *T*. *whipplei* (*35*). Only because we used cell cultures in shell vials were we able to culture *C*. *burnetii* and *F*. *tularensis* in our study. Similarly, additional cases with these fastidious organisms, as well as 1 case of infection with *T*. *whipplei*, were diagnosed because of systematic use of broad-range PCR on lymph nodes.

The cause of 351 cases of lymphadenopathy in this study could not be determined. Several reasons and limitations may explain this result. First, histologic data were obtained for only 23% of the lymph node specimens because most were sent to our center frozen or were too small. In 181 specimens, neoplasms may represent >25% of cases of suspected CSD. Thus, a similar proportion of neoplasms may be present in the remaining 605 specimens. For practical purposes, neoplasm can only be diagnosed by histopathologic analysis. Thus, lymph node excision is crucial in the diagnosis of malignant processes. Another limitation of our study was that we did not test for fungi or viruses that may also represent causes of lymphadenopathies. Mycobacterial infections in our study were diagnosed by culture and confirmed retrospectively by using a real-time quantitative PCR. We believe that the systematic use of real-time PCR for detection of mycobacteria will likely increase the percentage of such infections as causes of lymphadenopathies.

Previous studies reported that the percentage of undiagnosed cases varied from 17.2% to 39.7% (Table 3), and malignant processes were more common than infectious diseases. In more recent studies, percentages of lymph node specimens with malignant processes were lower (11.5%–17.3%), and infectious diseases were more common (17.7%–48.6%) (*1**,**8**,**36*) (Table 3).

We have showed that neoplasm could be clinically misdiagnosed as CSD. This finding was probably underestimated because we had previously analyzed lymph nodes only by culture and detection of fastidious organisms. Moreover, only fresh samples can be used in histologic analysis. Our results reemphasize that CSD may be misdiagnosed as neoplasm, and we believe that lymph node excision and histologic analysis are critical for accurate diagnosis.

We found that 13 *Bartonella*-positive patients (4.2%) had concurrent disease; 10 had mycobacteriosis (Figure 2), and 3 had neoplasm. These patients were older than those with CSD alone. However, neoplasm and mycobacterial infection was less common in patients with CSD than in those without CSD (p = 0.014, n = 181 patients). In the only report of coincidental CSD and neoplasm, Ridder et al. found 2 patients with squamous cell carcinoma and 2 patients with malignant B-cell lymphoma on the basis of high antibody titers to *B*. *henselae* (*1*). A high prevalence of *B*. *henselae*–specific antibodies in HIV-positive patients with generalized lymphadenopathy and patients with non-Hodgkin lymphoma has also been reported (*37*). Explanations for such associations are unknown, and the frequency of asymptomatic patients with CSD is not known. One may speculate that *Bartonella* infections produce more symptoms in patients with HIV infections, mycobacterial infections, or neoplasm or cause chronic infection in such cases.

In conclusion, lymph node excision and testing by histologic analysis are critical in detecting malignant processes and mycobacterial infections, even in patients found to have CSD by PCR. A diagnosis of CSD does not preclude other concurrent diseases, and their presence should routinely be tested by histologic analysis. In addition to testing pus samples or serologic analysis, biopsy specimens should be examined by a histologist, as recently proposed for patients with lymphadenopathy (*8**,**38**,**39*). Our study also demonstrates the advantage of specific target gene amplification compared with 16S rDNA gene amplification. Moreover, physicians should be aware that CSD can occur concurrently with neoplasm and mycobacteriosis, especially in adults >49 years of age.
